# The impacts of learning motivation, emotional engagement and psychological capital on academic performance in a blended learning university course

**DOI:** 10.3389/fpsyg.2024.1357936

**Published:** 2024-05-09

**Authors:** Yan Liu, Shuai Ma, Yue Chen

**Affiliations:** ^1^College of Primary Education, Hunan First Normal University, Changsha, China; ^2^Department of Agricultural Leadership, Education & Communications, Texas A&M University, College Station, TX, United States; ^3^College of Education, Hunan Agricultural University, Changsha, China

**Keywords:** psychological capital, learning motivation, emotional engagement, academic performance, blended learning

## Abstract

**Introduction:**

This study aims to explore the relationships among psychological capital, learning motivation, emotional engagement, and academic performance for college students in a blended learning environment.

**Method:**

The research consists of two studies: Study 1 primarily focuses on validating, developing, revising, and analyzing the psychometric properties of the scale using factor analysis, while Study 2 employs structural equation modeling (SEM) to test the hypotheses of relationships of included variables and draw conclusions based on 745 data collected in a university in China.

**Results:**

Findings revealed that intrinsic motivation, extrinsic motivation, emotional engagement, and psychological capital all impact academic performance. Extrinsic learning motivation has significant positive direct effects on intrinsic learning motivation, emotional engagement, and psychological capital. Intrinsic motivation mediates the relationship between extrinsic motivation and academic performance.

**Discussion:**

In future blended learning practices, it is essential to cultivate students’ intrinsic learning motivation while maintaining a certain level of external learning motivation. It is also crucial to stimulate and maintain students’ emotional engagement, enhance their sense of identity and belonging, and recognize the role of psychological capital in learning to boost students’ confidence, resilience, and positive emotions.

## 1 Introduction

From the perspective of social psychology, learning motivations, emotional engagement, and psychological capital originated from positive psychology, usually considered to impact academic performance positively. Learning motivation, especially intrinsic motivation, is positively correlated with academic outcomes measured by scores or grades (Schunk et al., 2008; Lazowski and Hulleman, 2016; Alkış and Taşkaya Temizel, 2018); Emotional engagement is considered to directly affect learning outcomes or indirectly affect by shaping behavioral and cognitive engagement (Raver, 2002; Bierman et al., 2008; Liew et al., 2008; Engels et al., 2021; Hasnine et al., 2023). Psychological capital, as an individual’s positive psychological state, leads to positive organizational behavior, and has a positive relationship with academic performance (Jafri, 2013; Khajavy et al., 2019; Carmona-Halty et al., 2021; King and Caleon, 2021; Slåtten et al., 2023). However, only limited research includes all three factors, although there is some study on interaction between two of these three factors.

In addition, after the sudden outbreak of COVID-19 in 2020, blended learning was accepted more widely, which may prepare students better for future work (Ravat et al., 2021). But the study of blended learning’s effect on the academic performance is scarce, while some researches show that blended learning has a more positive impact on academic performance than face-to-face and online learning, because learners hold significantly more positive attitudes toward blended learning (Cirak et al., 2018; Yu et al., 2022), these are determinative of the learning quality (Hasnine et al., 2023), the academic performance is influenced by emotional engagement (Gao et al., 2020). Regardless of gender, academic performance is mainly predicted by satisfaction levels, and the effects of perceived benefit and satisfaction level on academic performance are different between undergraduate students and postgraduate students in the blended context (Kobicheva et al., 2022). Perceived precision teaching (PPT) can effectively promote learning performance of college students in a blended environment (Yin and Yuan, 2021). Furthermore, in blended learning context, intrinsic and extrinsic motivation positively correlate with learning performance, especially the intrinsic motivation (Peng and Fu, 2021). And, there is a direct positive association between psychological capital and students’ learning engagement, and it may play a beneficial joint role in promoting academic performance in blended learning (Fu and Qiu, 2023). So far as we know, learning motivation, emotional engagement, and psychological capital correlate with blended learning performance to a certain extent, the purpose of this study is to:

1. Validate, develop and revise the psychometric properties of the scales of learning motivation, emotional investment, psychological capital in blended learning.
2. Analyses the interaction between learning motivations, emotional engagement and psychological capitals, and their jointly effect on blended learning performance.

## 2 Literature review

### 2.1 Emotional engagement

#### 2.1.1 Emotional engagement

Emotional engagement, in a brief, is students’ affective reactions to learning (Fredricks et al., 2004; Trowler and Trowler, 2010; Lester, 2013), including the belonging and the value of learning (Wang et al., 2011; Kahu, 2013; Wimpenny and Savin-Baden, 2013; Witkowski and Cornell, 2015) in the school context, and also the affective states or feelings such as happiness, interest, bore, sadness, and zest in the class context (Appleton et al., 2008; Wang et al., 2014; Manwaring et al., 2017; Pöysä et al., 2018). While emotions can be divided into both positive and negative emotions, emotional engagement can also be divided into emotional engagement and disaffection (Skinner et al., 2008; Dixson, 2010; Guthrie et al., 2012). Although the feasibility of using computer vision techniques to measure the emotional engagement by analyzing facial features is being discussed recently (Ashwin and Guddeti, 2020; Mehta et al., 2022; Hasnine et al., 2023), but it is a challenging task, so self-report is relatively mature measures technology (Fredricks and McColskey, 2012).

#### 2.1.2 Emotional engagement and academic performance

Studies have shown that emotional engagement correlated to educational outcomes, or positively related with academic performance and learning satisfaction (Connell et al., 1994; Fredricks et al., 2004; Halverson and Graham, 2019), there are a few studies on the emotional engagement’s impact on learning achievement, and some of them find that emotional engagement plays a vital role in predicting academic achievement (Raver, 2002; Bierman et al., 2008; Liew et al., 2008; Engels et al., 2021; Hasnine et al., 2023). Emotional engagement influences academic performance through behavioral shaping as a mediator (Geertshuis, 2019), or effect on learning achievement by the interaction with cognitive engagement (Liu et al., 2022).

#### 2.1.3 Emotional engagement and learning motivation

Some of the studies show that emotional engagement influences academic performance through motivation as a mediator (Skinner et al., 2009), motivation arises from many different sources, including emotions, the relations of affection and motivation are somewhat mixed (Daniels et al., 2009; Reeve, 2012), some factors of emotional engagement, such as self-acknowledgment, self-value, attitude, have a complex relationship with extrinsic and intrinsic motivation (Lee et al., 2022). In other words, emotional engagement can be positively predicted by students’ learning motivation (Ganotice et al., 2022).

### 2.2 Psychological capital

#### 2.2.1 Psychological capital

Psychological capital originated from positive psychology. It initially focuses on positive organizational behavior (Luthans and Youssef-Morgan, 2017). Psychological Capital (PsyCap) includes four constructs: hope, self-efficacy, resilience, and optimism (Luthans et al., 2007a,b). Psychological capital refers to a person’s positive psychological state of development. The four constructs are as follows: (1) self-efficacy refers to the confidence to take on and put in the required effort to succeed when facing challenging tasks; (2) optimism refers to making a positive attribution about success not only now but also in the future; (3) hope refers to persevering toward objectives and, when required, redirecting paths to goals to succeed; and (4) resilience refers to sustaining and bouncing back and even beyond to attain success when beset by problems and adversity (Luthans et al., 2007a,b).

#### 2.2.2 Psychological capital and academic performance

Studies have suggested a positive relationship between psychological capital and academic performance (Jafri, 2013). Psychological capital affects academic performance (Carmona-Halty et al., 2021; Slåtten et al., 2023). School psychological capital was associated with optimal academic outcomes (King and Caleon, 2021). Learners’ psychological capital can significantly and positively predict second language achievement (Khajavy et al., 2019).

### 2.3 Learning motivation

#### 2.3.1 Motivation and learning motivation

Researchers have defined the motivation from different perspectives. Motivation guides the direction, intensity and persistence of performance behavior, which can be either intrinsic (guided by a person’s inner beliefs and choices) or extrinsic (driven by some external sources other than the person himself) type (Cerasoli et al., 2014; Deci et al., 2017). From the perspective of cognitive psychology, motivation is an internal mental process or internal motivation that guides, stimulates and sustains an individual’s activities through goals or objects (Pintrich and Schunk, 1996). More specifically, the conceptions of extrinsic motivation include orientations of ward money, recognition, competition, and the deference to other people, while intrinsic motivation includes challenge, enjoyment, personal enrichment, interest, and self-determination (Amabile et al., 1994).

Learning motivation is the practical application of motivation theory in school learning (Slavin, 2005). How to stimulate, transform, and maintain students’ learning motivation is an essential topic in the field of motivation study. Many previous motivation studies have been investigated on the set of school learning. However, the earlier studies mainly have been conducted in traditional learning environments.

#### 2.3.2 Learning motivation and academic performance

Learning motivation positively affects students’ course grades (Schunk et al., 2008). Students who are motivated to learn will have more tremendous success than those who are not (Hodges, 2004). In addition to learning more, the students who intrinsically motivated are proved to get higher score on standardized achievement tests, persist longer, and produce higher quality work (Fredricks and McColskey, 2012; Lazowski and Hulleman, 2016). Among the six motivation factors measured by MSLQ, intrinsic motivation has the most significant influence on academic performance (Alkış and Taşkaya Temizel, 2018). Some research shows that in a Blended Learning environment, motivational factors positively affect the students’ overall grades (Ramirez-Arellano et al., 2019).

#### 2.3.3 Learning motivation and psychological capital

The relationship between learning motivation and psychological capital has been a research focus recently. Despite many past studies examining the relationship between learning motivation and psychological capital in traditional learning environments, research in the blended learning environment has been relatively rare. Previous studies have found that psychological capital can predict motivation (Khajavy et al., 2019; King and Caleon, 2021; Ali et al., 2022). Psychological capital and intrinsic motivation play a mediating role in self-rated mindfulness and study engagement (Ali et al., 2022). And school psychological capital positively predicted optimal motivation (King and Caleon, 2021). Psychological capital plays a significant predictive role in L2 motivational self-system (Khajavy et al., 2019). Psychological capital was positively related to motivation in a military college (LaRocca et al., 2023). There is a positive relationship between positive psychological capital and motivation perceptions (Ahmet et al., 2022). Some studies also indicated a bidirectional relationship between motivation and psychological capital, suggesting motivation can impact psychological capital. For example, work motivation contributes significant variance in psychological capital (Hasnain et al., 2017).

## 3 The present study: hypotheses and proposed model

**Figure 1:**
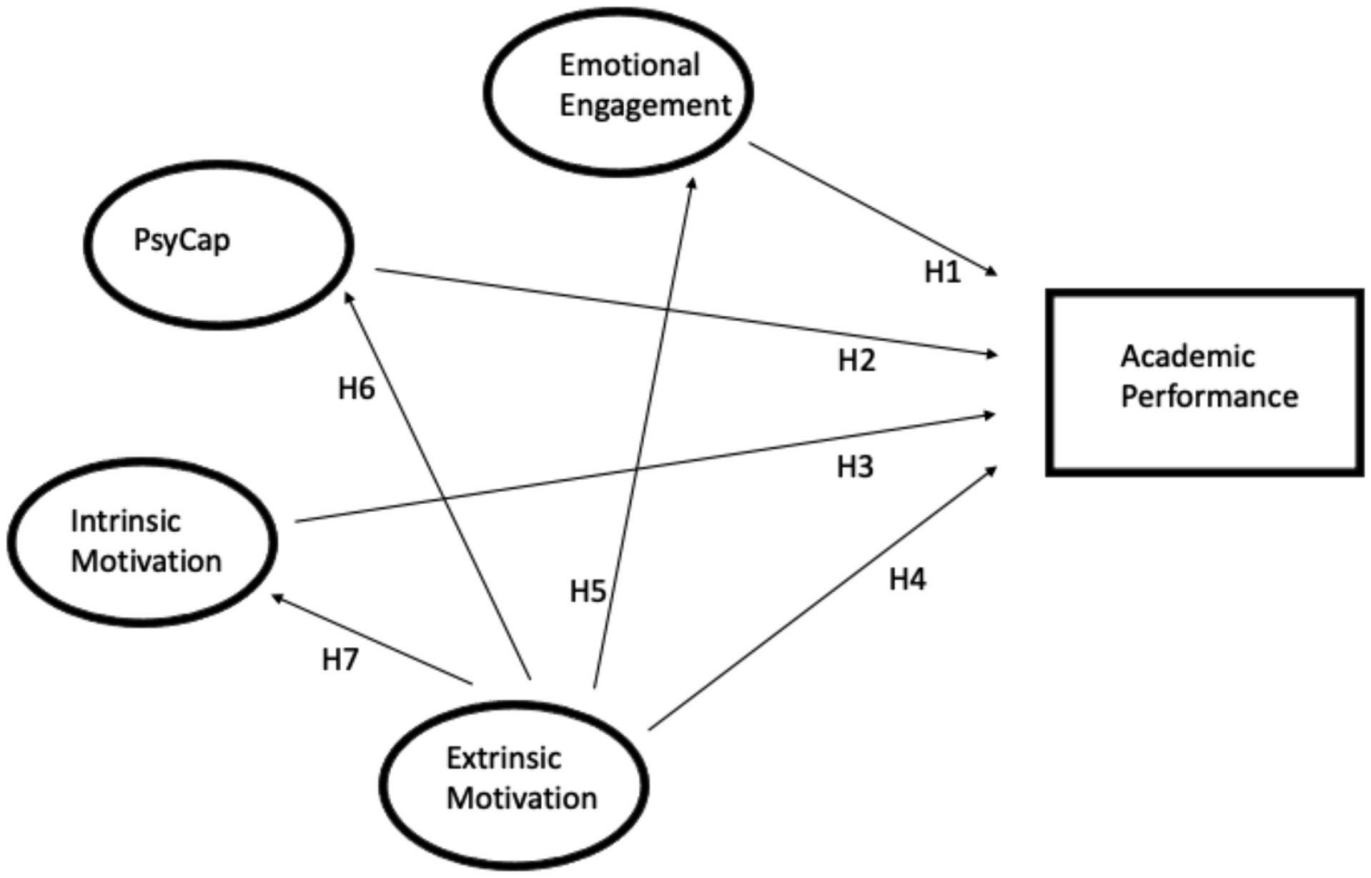
Proposed model.

The present study aims to investigate the relationship among emotional engagement, psychological capital, intrinsic motivation, extrinsic motivation, and academic performance. Figure 1 demonstrates the proposed model. The proposed hypothesis in the model is as follows based on a review of previous literature:

1. There is a direct positive relationship between Emotional Engagement and Academic performance. (H1)

2. There is a direct positive relationship between Psychological Capital and Academic performance. (H2)

3. There is a direct positive relationship between Intrinsic Motivation and Academic performance. (H3)

4. There is a direct positive relationship between Extrinsic Motivation and Academic performance. (H4)

5. Extrinsic Motivation can positively predict Emotional Engagement. (H5)

6. Extrinsic Motivation can positively predict Psychological Capital (PsyCap). (H6)

7. Extrinsic Motivation can positively predict Intrinsic Motivation. (H7)

## 4 Method

### 4.1 Participants and procedures

#### 4.1.1 Participants

This study employs the convenience sampling method for the selection of samples. The survey participants encompass students who were enrolled in the “Educational Policies and Regulations and Teachers’ Professional Ethics” course during the first semester of 2023 at Hunan First Normal University, situated in China. Most of these students are in their sophomore year and pursuing an education major. This study first recruited 253 participants for a pilot study to validate and develop the instrument for study 1. The total cohort consists of 812 students. After deleting samples that had missing information, 745 participants were used for data processing for study 2. Notably, the “Educational Policies and Regulations and Teachers’ Professional Ethics” course is designed as a pedagogical training course for aspiring teachers. The instructional approach for this course integrates both online and offline components. This blended mode of instruction has been consistently implemented across four semesters, culminating in establishing a notably stable teaching framework. A comprehensive overview of the subjects’ demographic information is presented in Table 1.

**Table 1:** Demographic information of participants.

Variables	Groups	Number	Frequency (%)
Sex	Female	625	83.89
	Male	120	16.11
Previous blended learning experience	Never	68	9.13
	One semester	72	9.66
	Two semesters	115	15.44
	Three semesters	122	16.38
	Four semesters or more	368	49.40
Subject	Liberal arts	445	59.73
	Science	167	22.42
	Engineering	6	0.81
	Others	127	17.05
Preferred learning style	Online	126	16.91
	Offline	159	21.34
	Blended	434	58.26
	Not sure	26	3.49

#### 4.1.2 Procedures

This study mainly uses the questionnaire which consists of three scales: emotional engagement, psychological capital, and learning motivation. After compiling the questionnaire, we fully consulted the respondents’ consent to participate. Students willing to participate in the questionnaire fill in the questionnaire through the online questionnaire platform called Wenjuanxing. Before the formal investigation, this study conducted a small-scale pilot questionnaire test. After analyzing the reliability and validity of these test samples, the questionnaire’s items were modified by deleting some items to modify the final questionnaire. As for the missing data, this study deleted the data according to the respondents’ situation, such as the answer time being too short, the answer being incomplete, etc. The survey was adopted and developed in English and then translated into Chinese to maximize the understanding of questionnaire before distributing.

### 4.2 Measures

#### 4.2.1 Emotional engagement scale (EES)

The Emotional Engagement Scale was a researcher-developed questionnaire with 10 items which was based on Skinner et al. (2009), Wang et al. (2014), Maroco et al. (2016) and Pöysä et al. (2018). The 10 items are E1) I feel happy in the class. E2) I feel proud in the class. E3) I feel excited in the class. E4) I feel interested in the class. E5) I have fun in the class. E6) I discuss with my colleagues about this class in a positive way possible way. E7) I discuss with our teacher about this class in a positive way. E8) Our university has provided good support for learning this course. E9) In general, I feel like a real part in this class. E10) it is worthful to learn this class. But final scale deleted five items (E6, E7, E8, E9, E10) based on the factor analysis results, which is similar to Wang et al. (2014). In study 2, Emotional engagement refers to the students’ positive affective reactions in the class, including enthusiasm, pride, zest, interest, and enjoyment, which are measured by remained five items (E1, E2, E3, E4, E5) with 5-point Likert scale where 5 indicating the strongest level of emotional engagement.

#### 4.2.2 Psychological capital scale (PCS)

The scale of Psychological Capital was adopted from Luthans et al. (2007a,b). The 12 items originally consisted of four constructs: hope, self-efficacy, resilience, and optimism. The 12 items are: P1) I feel confident to participate in the relevant meeting/competition on behalf of the class. P2) I feel confident contributing to suggestions at school to my teachers. P3) I feel confident presenting information to a group of classmates at school. P4) If I should find myself in a jam at school, I could think of many ways to get out of it. P5) Right now, I see myself as being successful at school. P6) I can think of many ways to reach my current goals at school. P7) At this time, I am meeting the goals that I have set for myself at school. P8) I can be “on my own,” so to speak, at school if I have to. P9) I usually take stressful things at school in stride. P10) I can get through difficult times at school because I’ve experienced difficulty before. P11) I always look on the bright side of things regarding my coursework. P12) I’m optimistic about what will happen to me in the future as it pertains to school. The scale employs a 5-point Likert scale, with 5 indicating the strongest level of Psychological Capital. The PCS includes Efficacy (P1-P3), Hope (P4-P7), Resilience (P8-P10) and Optimism (P11-P12). Three items (P1, P11, P12) were deleted and nine items remained based on factor analysis results to be used in Study 2.

#### 4.2.3 Learning motivation scale (LMS)

The Learning Motivation Scale used in this study primarily draws from Amabile et al.’s (1994) motivation scale, which has been adjusted to fit the educational context and to suit better the measurement of college students’ motivation to learn. The learning motivation scale consists of 10 items, five dedicated to intrinsic motivation and five to extrinsic motivation. The 10 items are M1) I hope that studying this course will provide me with opportunities to enhance my knowledge and skills. M2) I enjoy tackling problems that are completely new to me in this course. M3) I will be content as long as this course helps me improve, regardless of the outcome. M4) Being able to learn something I’m interested in while studying this course is important to me. M5) The more difficult problems I encounter in this course, the more I enjoy trying to solve it. M6) I take this course seriously as it’s a required/teacher education course. M7) Achieving good grades is a significant goal in my study of this course. M8) The more difficult problems I encounter in this course, the more I enjoy trying to solve it. M9) Even if I perform well but my parents, teachers, and classmates are unaware, studying this course loses meaning. M10) Gaining approval and recognition from my parents, teachers, and classmates motivates me to work hard in this course. After undergoing reliability and validity testing and subsequent analysis, three items (M2, M5, M8) were deleted and the questions were refined and ultimately reduced to seven items to be used for Study 2. These items comprise four questions related to intrinsic motivation (M1, M3, M4, M6) and three related to extrinsic motivation (M7, M9, M10). The scale employs a 5-point Likert scale, where a higher score reflects a stronger level of motivation.

#### 4.2.4 Academic performance

To evaluate students’ course learning effectiveness, this study directly relies on the academic performance within the course. This encompasses both grades from homework/assignments throughout the semester and final grades, with each component contributing equally at 50%.

### 4.3 Data collection and analysis

This study primarily employs three scales: the Learning Motivation Scale, the Psychological Capital Scale, and the Emotional Engagement Scale. These scales are utilized to assess students’ learning motivation, psychological capital, and emotional investment, respectively. Additionally, the academic performance within the “Educational Policies and Regulations and Teachers’ Professional Ethics” course at Hunan First Normal University is leveraged as a measure of students’ course learning outcomes. The demographic information of the participants is also reported. Students’ course academic performance is evaluated through their academic grades, which are calculated using a percentage system. In the data analysis phase, SPSS was used for analyzing reliability and validity for study 1. Kaiser-Meyer-Olkin and Bartlett’s spherical test was used for examining the assumption, factor analysis, consisting exploratory factor analysis (EFA) and Confirmatory factor analysis (CFA), was employed to exam the psychometric property of the instrument. As for study 2, descriptive analysis and correlation analysis were run in STATA (18.0). Mplus software (VERSION 8.1) was employed for CFA, Structural Equation Modeling (SEM) and mediation analysis to investigate the model and relationship among factors.

## 5 Study 1

### 5.1 Construct validity and reliability

Although the first measures of learning motivation, emotional engagement, and psychological capital were developed from the classic scales, which were subjected to a reliability and validity test, it is needed to be validated among the Chinese population. So, a revalidation of EEPCLM (the whole scale) pre-test with 253 samples was made. Firstly, this study assesses its appropriateness. Kaiser-Meyer-Olkin measure of sampling adequacy (KMO =0.966) and Bartlett’s spherical test (χ^2^ = 15823.855, *p* < 0.001) indicate that the assumptions were fulfilled and this questionnaire is suitable for exploratory factor analysis (EFA). Secondly, factor analysis was performed with a specified number four. The analysis results were subjected to item deletion. Five items (E6, E7, E8, E9, E10) from Emotional Engagement Scale were deleted. Three items, namely P1, P11, and P12 from Psychological Capital Scale were deleted. Three items (M2, M5, M8) from Learning Motivation Scale were deleted. After seven iterations of the initial factor load matrix, the rotated factor load matrix was obtained, which meets the theoretical framework and statistical requirements. Thirdly, a further factor analysis was conducted on the remaining 21 items, and it was found that the eigenvalues of all four factors were greater than 1. The cumulative contribution rate of interpretable variance is 79.11%, and the standardized factor loadings for the four sub-dimensions of the model range from 0.529 to 0.870. According to Brown (2015), the factor loadings are suggested to be higher than 0.5, and the Table 2 indicates that the extracted factors are identifiable and construct validity of the adapted scale is of high quality. According to Table 2, there are seven items of LMS (four items of intrinsic and three items of extrinsic learning motivation), five items of EES, and nine items of PCS. The Confirmatory factor analysis (CFA) results show that all goodness-of-fit indices are acceptable (χ2 / df = 6.546, *p* > 0.05, RMSEA = 0.086, GFI = 0.850, AGFI = 0.811, NFI = 0.925, RFI = 0.914, IFI = 0.936, TFI = 0.926, CFI = 0.936), which indicated that this 21-item instrument has a good model fit when tested with the data from the 253 pre-tested learners.

**Table 2:** Standardized CFA solutions for the EEPCLM.

Items	Factor loading			
	PCS	EES	In-LMS	Ex-LMS
Item 1 (E1)		0.809		
Item 2 (E2)		0.801		
Item 3 (E3)		0.820		
Item 4 (E4)		0.822		
Item 5 (E5)		0.790		
Item 6 (P2)	0.733			
Item 7 (P3)	0.762			
Item 8 (P4)	0.740			
Item 9 (P5)	0.739			
Item 10 (P6)	0.745			
Item 11 (P7)	0.721			
Item 12 (P8)	0.669			
Item 13 (P9)	0.684			
Item 14 (P10)	0.650			
Item 15 (M1)			0.738	
Item 16 (M3)			0.691	
Item 17 (M4)			0.731	
Item 18 (M6)			0.742	
Item 19 (M7)				0.529
Item 20 (M9)				0.870
Item 21 (M10)				0.799
Eigenvalue	5.777	4.617	3.925	2.294
Contribution rate %	27.509	21.985	18.691	10.924
Accumulated contribution rate %	27.509	49.494	68.185	79.108

PCS, Psychological Capital Scale; EES, Emotional Engagement Scale; In-LMS, Intrinsic Motivation Scale; Ex-LMS, Extrinsic Motivation Scale.

This study uses the convergent and discriminant validity techniques to measure the construct validity. Firstly, the convergent validity refers to the degree to which the variables measuring the same construct are associated with each other and the construct they belong (Raykov and Marcoulides, 2011). In order to provide convergent validity, it is recommended that the item loadings obtained by each construct is higher than 0.05, and it is recommended that the average variance extracted by each structure is equal to or higher than 0.05, and less than Cronbach Alpha and composite reliability values (Fornell and Larcker, 1981; Hair et al., 2010). According to Nunnally and Bernstein (1994), Cronbach Alpha is recommended to be higher than 0.7. Table 3 shows the extracted average variance, Cronbach Alpha, and composite reliability values. According to Table 3, the convergent validity of the scale is satisfactory, and these results prove that the reliability of the total scale is sufficient.

**Table 3:** The average variance values and the reliability coefficients obtained for the constructs.

	PCS	EES	In-LMS	Ex-LMS
Average variance extracted	0.7211	0.8179	0.7029	0.597
Composite reliability	0.9587	0.9573	0.9036	0.8145
Cronbach’s alpha	0.957	0.958	0.898	0.795

PCS, Psychological capital; EES, Emotional engagement; In-LMS, Intrinsic Motivation; Ex-LMS, Extrinsic Motivation.

Secondly, discriminant validity refers to the degree to which each potential variable in the model is distinguished from the others (Farrell, 2009). It requires that the square root of the average variance interpreted for each construct be no less than the value that each construct relates to the other constructs (Fornell and Larcker, 1981; Hair et al., 2010). Table 4 shows the correlation of structures in the scale. According to Table 4, the correlations among the constructs are moderate and significant.

**Table 4:** Results of discriminate validity.

	EES	PCS	In-LMS	Ex-LMS
EES	0.904			
PCS	0.434***	0.849		
In-LMS	0.342***	0.338***	0.838	
Ex-LMS	0.385***	0.504***	0.283***	0.773

PCS, Psychological capital Scale; EES, Emotional engagement Scale; In-LMS, Intrinsic Motivation Scale; Ex-LMS, Extrinsic Motivation Scale. The values (in bold) on the diagonal are the square root of AVE values. The values below the diagonal are the correlations between the constructs. *p < 0.05, **p < 0.01, ***p < 0.001.

## 6 Study 2

### 6.1 Impacts of variables on academic performance

#### 6.1.1 Preliminary analysis

As for academic performance, it ranges from 39.2 to 92.43 with the mean of 77.34 and the standard deviation of 6.75. Analysis of the correlations between the variables utilized in the survey has supported this study’s hypotheses substantially. This is demonstrated in Table 5, where the statistically significant relationships between emotional engagement, extrinsic motivation, intrinsic motivation, and academic performance are evident (*p* < 0.05) as well as the relationship among variables (*p* < 0.001).

**Table 5:** Descriptive statistics, internal consistency reliability, and correlations among the variables.

	1	2	3	4	5
1. Psychological capital					
2. Emotional engagement	0.73***				
3. Extrinsic motivation	0.65***	0.46***			
4. Intrinsic motivation	0.75***	0.72***	0.49***		
5. Academic performance	0.04	0.08*	−0.10*	0.08*	
Mean	33.33	19.90	10.21	16.25	77.34
SD	7.10	4.08	2.84	2.78	6.75
Min	9	5	3	4	39.2
Max	45	25	15	20	92.43
Cronbach’s alpha	0.96	0.96	0.80	0.91	

*p < 0.05, **p < 0.01, ***p < 0.001.

#### 6.1.2 Measurement model

The model incorporates four factors: psychological capital, emotional engagement, intrinsic motivation, and extrinsic motivation. Confirmatory Factor Analysis (CFA) was employed to evaluate the goodness of fit between the proposed model and the observed data. This study considered various fit indices, including the root mean square error of approximation (RMSEA) with a threshold value of 0.08 (Hu and Bentler, 1999), standardized root mean squared residuals (SRMR) with a threshold value of 0.08 (Byrne, 1994), and the comparative fit index (CFI) with a threshold value of 0.90 (Byrne, 1994). Initially, a measurement model was established with *χ2* (204) = 1253.822, *p* < 0.001, RMSEA = 0.083, SRMR = 0.065, CFI =0.934, revealing an inadequate fit. Subsequently, the model was respecified by allowing correlation between error terms of specific items: Item 6 and Item 7, item 13 and Item 14, Item 18 and Item 19, and Item 20 and Item 21. Following these adjustments, the confirmatory factor analysis of the revised measurement model demonstrated a good fit, with *χ*^2^ (179) = 745.488, *p* < 0.001, RMSEA = 0.063, CFI = 0.962, SRMR = 0.041.

#### 6.1.3 Structural model

A Structural Equation Modeling was constructed with extrinsic motivation as the predictor and psychological capital, intrinsic motivation, and extrinsic motivation as outcomes. Meanwhile, the model with psychological capital, emotion, intrinsic motivation, and extrinsic motivation as predictors and academic performance as outcomes was evaluated. Psychological capital, emotion, intrinsic motivation, and extrinsic motivation were proposed as latent variables. In the structural model, the model demonstrated a good fit (*χ*^2^ = 859.925 (199), *p* < 0.001, RMSEA = 0.067, CFI = 0.958, SRMR = 0.043).

**Figure 2:**
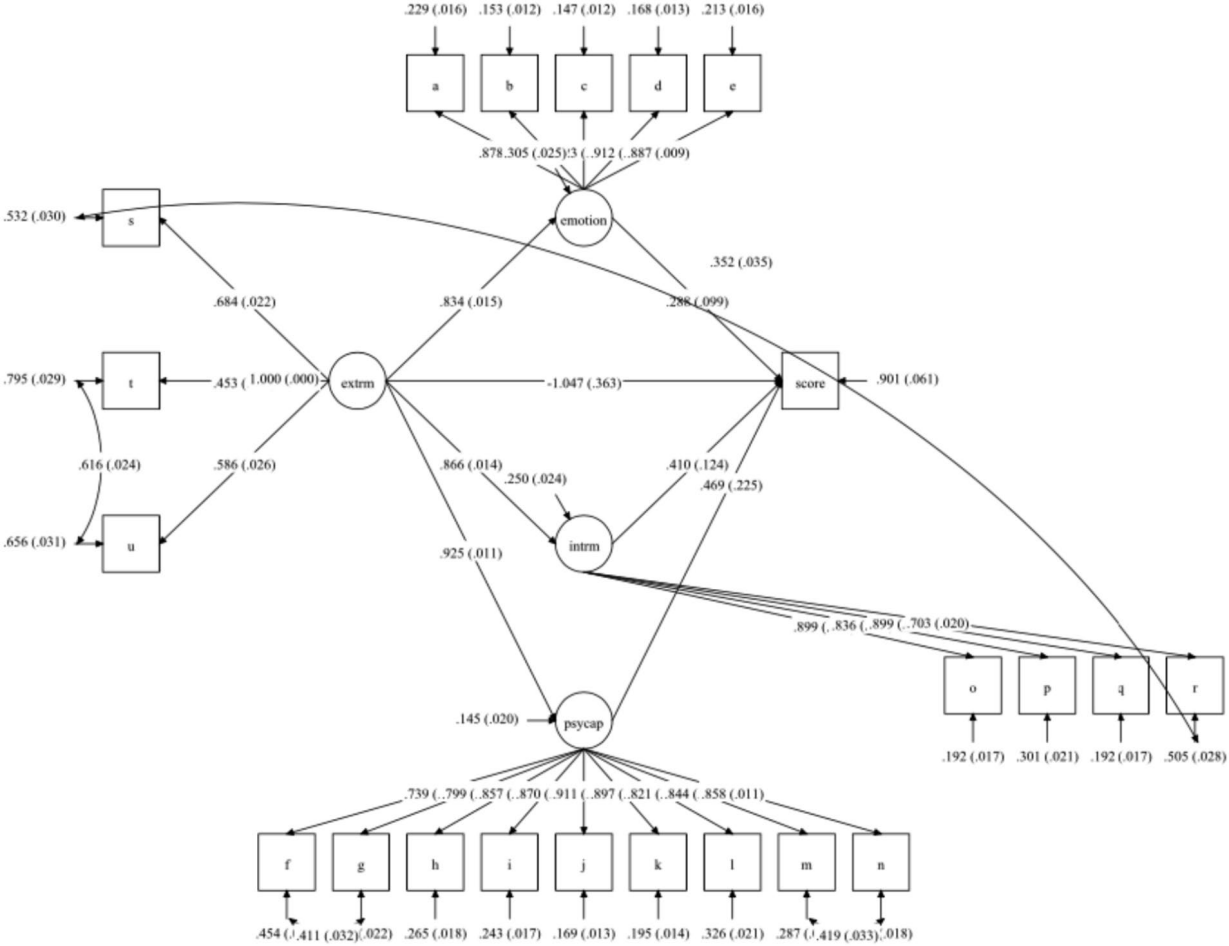
Path diagram of the structural model using SEM. Emotion, Emotional Engagement; score, Academic Performance; Intrm, Intrinsic Motivation; extrm, Extrinsic Motivation; psycap, Psychological Capital.

Figure 2 demonstrated the path diagram of structural model. The line represents a significant path and the arrow indicated the direction. The coefficient next to the line is the *β* coefficient. Table 6 displays this model with a standardized regression coefficient. The significant direct path coefficients were: 1) Emotional engagement positively predicted academic performance (*β* = 0.29, *p* < 0.05), confirming H1, 2) Psychological capital positively predicted academic performance (*β* = 0.47, *p* < 0.05), therefore H2 was supported, 3) From intrinsic motivation to academic performance (*β* = 0.41, *p* < 0.05). H3 was supported, 4) From extrinsic motivation to academic performance (*β* = −1.05, *p* < 0.05). H4 was not supported, 5) From extrinsic motivation to emotional engagement (*β* = 0.83, p < 0.05). H5 was supported, 6) From extrinsic motivation to psychological Capital (*β* = 0.93, *p* < 0.05). H6 was supported, and 7) From extrinsic to intrinsic motivation (*β* = 0.97, p < 0.05). H7 was supported.

**Table 6:** Hypothesis results.

Hypothesis	Direct effect	β	*p*-value
H1	Emotional Engagement→Academic performance	0.29	< 0.05
H2	Psychological Capital→Academic performance	0.47	< 0.05
H3	Intrinsic Motivation→Academic performance	0.41	< 0.05
H4	Extrinsic Motivation→Academic performance	−1.05	< 0.05
H5	Extrinsic Motivation→Emotional Engagement	0.83	< 0.05
H6	Extrinsic Motivation→Psychological Capital	0.93	< 0.05
H7	Extrinsic Motivation→Intrinsic Motivation	0.97	< 0.05

#### 6.1.4 Test for mediation

As a negative coefficient was observed from Extrinsic Motivation to Academic performance from the SEM result, indicating a negative association between extrinsic motivation and academic performance. Further analysis is needed to further investigate the relationship between the motivation and academic performance. Based on researcher’s particular interest in how motivation plays a role, a mediation analysis was thus employed in the model to see if Intrinsic Motivation mediates the relationship between Extrinsic Motivation and Academic performance. The total indirect effect estimate is 2.184 with *p* < 0.05, indicating that the total indirect effect is statistically significant (Table 7). The results indicate a significant total mediated effect of Extrinsic Motivation on Academic Performance through the mediator Intrinsic Motivation.

**Table 7:** Mediator analysis.

Mediation path	Total effects	Indirect effect			Direct effect		
		Estimate	S.E.	*p*-value	Estimate	S.E.	*p*-value
ExtrM→IntrM→AP	0.532	2.184*	0.930	0.02	−1.652	1.082	0.13

*p < 0.05. ExtrM, Extrinsic motivation; IntrM, Intrinsic Motivation.

## 7 Discussion and suggestion

This study investigates the relationship between college students’ learning motivation, emotional engagement, psychological capital, and academic performance in the blended learning context. It aims to discern which factors (learning motivation, emotional engagement, and psychological capital) exert the most pronounced influence on students’ academic performance. Prior research has yet to delve into this specific relationship within the blended learning framework. This study revealed the following findings: 1). The intrinsic motivation, emotional engagement, and psychological capital positively and directly impact their academic performance significantly in higher education in a blended learning context, 2) The emotional engagement of college students directly and positively influences their academic performance significantly within the blended learning environment, 3) Extrinsic motivation directly impacts intrinsic motivation, emotional engagement, and psychological capital significantly in higher education in blended learning environment, and 4) Intrinsic motivation mediates the relationship between extrinsic motivation and academic performance significantly. The total effect of extrinsic motivation on academic performance is positive.

These findings reveal a noteworthy positive correlation between intrinsic learning motivation and learning outcomes, aligning with prior research (Fredricks and McColskey, 2012; Lazowski and Hulleman, 2016). Similarly, the observed positive impact of emotional engagement on college students’ learning outcomes is consistent with earlier conclusions from research (Raver, 2002; Bierman et al., 2008; Liew et al., 2008; Engels et al., 2021; Hasnine et al., 2023). In the blended learning environment, the psychological capital of college students has a significant impact on the learning effect of college students, which is in alignment with previous research conclusions (Jafri, 2013). This study shows that in a blended learning environment, college students’ emotional engagement is positively correlated with academic performance, and the higher the emotional engagement of students, the better their academic performance. This is consistent with other research findings (Liu et al., 2022) in blended and traditional learning settings. However, this study found a direct negative relationship between extrinsic motivation and academic performance. Such a result may be related to the learning context of college students. In adult education, students are independent, self-regulated learners. Too much extrinsic pressure from outside would make adult learners feel like their responsible adult learners are rejecting them; thus, a subconscious resentment and resistant psychological state would appear to affect their learning behavior (Merriam and Bierema, 2013). Another possible reason might be the different learning context from previous research. This research is based on the blended learning environment, while the previous research is based on the traditional face-to-face learning environment. Nearly half of the participants in this study reported that they had four semesters’ blended learning experience and their prefered learning style is blended learning setting suggesting their familarity and comfortable of learning in a blended context. Thus, the characteristics of the selected participants associated with rich blended learning experience might also contribute to the academic performance. Another interesting finding is the mediation role of intrinsic motivation between extrinsic motivation and academic performance. Previous studies have found motivation as a mediator. For example, Autonomous motivation mediates the relationship between individual psychological capital and innovative work behavior (Blasco-Giner et al., 2023). Intrinsic motivation was measured as a significant mediator between psychological capital and study engagement (Siu et al., 2014). This study contributed to the literature by broadening intrinsic motivation as the mediator between extrinsic motivation and academic performance. This study confirmed the essential role of intrinsic motivation.

Building upon the above research findings, this study proposes the following recommendations for enhancing blended learning practices within university classrooms: Firstly, emphasize enhancing intrinsic and proper extrinsic motivation: Concentrate on fostering students’ internal drive for learning. This study reveals a significant positive correlation between intrinsic learning motivation and academic performance in a blended learning environment. In contrast, intrinsic motivation is the mediator between extrinsic motivation and academic performance, resulting in a positive total effect from extrinsic motivation to academic performance. Without the mediating effect of intrinsic motivation, extrinsic motivation might lead to a negative effect on academic performance in certain context. This signifies that, in the context of blended learning in universities, students’ internal motivation emerges as a vital factor in enhancing academic performance. As a previous study shows that an explicitly strong and significantly positive main effect is found between intrinsic motivation and creative/innovative performance (Fischer et al., 2019). Thus, educational institutions need to create an environment that supports and nurtures students’ intrinsic learning motivation. Maintaining proper extrinsic motivation would benefit students by impacting their intrinsic motivation, emotional engagement, and psychological capital. In the context of blended teaching practices, educators should employ all kinds of methods and strategies to increase the intrinsic motivation of students, including adopting student-centered teaching methodologies, amplifying online discussions and interactions, elevating the quality of offline classroom experiences, and thereby augmenting students’ engagement and the overall effectiveness of blended instruction. To this end, attending to students’ emotions, attitudes, and values is crucial, ensuring a comprehensive educational experience. Students should cultivate a healthy perspective on learning. This entails developing a genuine interest in course material and regarding learning as a means of personal growth rather than a mere pursuit of high grades. Moreover, a departure from conventional learning methods, such as rote memorization and passive listening, is advised. Instead, students should embrace more proactive approaches, such as autonomous learning, inquiry-based exploration, and participatory engagement, which are integral to the principles of blended learning. Secondly, cultivate and sustain emotional engagement to foster a sense of belonging. Learning engagement includes cognitive engagement, emotional engagement, and behavioral engagement. This study only focuses on emotional engagement. And this study indicated the impact of emotional engagement on learning outcomes. Therefore, we should pay attention to students’ emotional investment in blended teaching. Specifically, in blended teaching, teachers, as important guides for students, should focus on improving students’ learning interest, stimulating their enthusiasm, making them feel proud, enhancing their passion, then make them really enjoy the class. For example, we can use the advantages of online teaching to strengthen and pay attention to the construction of online learning communities, promote emotional communication and exchanges between teachers and students, and enhance student interaction and discussion. At the same time, colleges and universities should strive to create a good, blended learning environment for students, provide sufficient support for students learning, improve students’ blended learning experience, shorten the psychological distance between teachers-students and students-students, because there is a study indicating that an improved educational environment can lead to an increased sense of well-being for students (Tripon et al., 2023). Thirdly, emphasizing the role of students’ psychological capital in learning, enhancing students’ self-confidence, resilience, and positive emotions. This study indicates that psychological capital can predict their academic performance among university students in a blended learning environment. To elaborate further, university students with higher levels of psychological capital in blended learning demonstrate greater self-efficacy, resilience, and positive emotions during the learning process. They are more willing to challenge high goals, leading to better academic performance. Therefore, in the context of blended teaching at the university level, it is crucial to recognize the significant role of students’ psychological capital in their learning. Specifically, as educators, we should emphasize nurturing students’ strong self-confidence in overcoming challenges. This can be achieved by creating online and offline classroom environments and platforms that facilitate improved self-efficacy. Encouraging students to express themselves boldly and enhancing their self-confidence is essential. Setting challenging online learning tasks also encourages students to think independently and solve problems, thereby developing their resilience and problem-solving skills. Furthermore, teachers should foster emotional connections with students beyond the classroom, using online advantages and platforms to engage in meaningful communication. This allows teachers to gain timely insights into students’ psychological and emotional dynamics and provide appropriate guidance. Based on this, teachers can adjust and optimize their teaching methods to enhance classroom teaching effectiveness.

For researchers, future research can conduct similar research in different subjects and cultural settings to validate the research findings on the impact of psychological capital, emotional engagement, and motivation on academic performance. The interrelationship between those variables can be further investigated using different models. Experimental design can also be used to validate the findings. The mechanism of how those variables interact with each other and how the variables impact learning can be further explored in educational psychology using interviews or qualitative methods.

## 8 Conclusion and limitation

This study aims to investigate the impacts of learning motivation, emotional engagement and psychological capital on academic performance in the blended learning university courses. Prior research has yet to delve into this specific relationship within the blended learning framework. This study originally revealed that there is a direct positive relationship between emotional engagement and academic performance, as well as psychological capital and academic performance in a blended learning context, while there is also a direct negative relationship between extrinsic motivation but a total positive effect on extrinsic motivation and academic performance due to mediator intrinsic motivation. Therefore, this study proposes that in the blended learning university courses we should emphasize enhancing intrinsic and proper extrinsic motivation; Cultivate and sustain emotional engagement to foster a sense of belonging; And emphasize the role of students’ psychological capital in learning, enhancing students’ self-confidence, resilience, and positive emotions.

Like most other research, this study mainly adopts self-reported methods with a closed-ended questionnaire for data collection rather than advanced technological means, such as computer vision techniques analyzing facial features. This data collection mode prevented participants from fully expressing their thoughts and feelings towards the variables we investigated, so there may be some subjectivity in the data. Secondly, due to the fact that participants from study 1 answered the original complete scale while participants from study 2 answered the scale after the validated scale with several items that were later removed, the researchers included the data of 253 students who took part in study 1 and also analyzed it in study 2. Thirdly, this study was representative of 745 students from a provincial university in Central China, while learning motivation, emotional engagement, and psychological capital may be different among students from different-level colleges such as key universities or vocational colleges, not to mention the learning performance. Therefore, we advise that generalization of these findings should be done cautiously. We encourage the replication of this study with other students from top universities in other geographical locations and more education experiments with an experimental group and control group with a mixed research method, including using computer vision techniques to analyze facial features.

## Data availability statement

The original contributions presented in the study are included in the article/supplementary material, further inquiries can be directed to the corresponding author.

## Ethics statement

Ethical approval was not required for the studies involving humans because ethical review and approval was not required for the study on human participants in accordance with the local legislation and institutional requirements (China). The studies were conducted in accordance with the local legislation and institutional requirements. Written informed consent for participation was not required from the participants or the participants' legal guardians/next of kin in accordance with the national legislation and institutional requirements because written informed consent from the participants was not required to participant in this study in accordance with the national legislation and the institutional requirements (China).

## Author contributions

YL: Conceptualization, Investigation, Project administration, Writing – original draft. SM: Writing – original draft, Formal analysis, Methodology. YC: Writing – original draft, Validation.

## References

[ref1] Ahmet R. Ö. Sinan Y. Nurullah B. (2022). Relationship between teachers' positive psychological capital, organisational commitment and motivation levels. Arch. Pharm. Chem. (Kbh) 18:1. doi: 10.26634/jsch.18.2.19257

[ref2] Ali M. Khan A. N. Khan M. M. Butt A. S. Shah S. H. H. (2022). Mindfulness and study engagement: mediating role of psychological capital and intrinsic motivation. J. Prof. Cap. Community 7, 144–158. doi: 10.1108/JPCC-02-2021-0013

[ref3] Alkış N. Taşkaya Temizel T. (2018). The impact of motivation and personality on academic performance in online and blended learning environments. Educ. Technol. Soc. 21:37, Available at: https://www.jstor.org/stable/26458505

[ref4] Amabile T. M. Hill K. G. Hennessey B. A. Tighe E. M. (1994). The work preference inventory: assessing intrinsic and extrinsic motivational orientations. J. Pers. Soc. Psychol. 66, 950–967. doi: 10.1037/0022-3514.66.5.950, PMID: 8014837

[ref5] Appleton J. J. Christenson S. L. Furlong M. J. (2008). Student engagement with school: critical conceptual and methodological issues of the construct. Psychol. Sch. 45, 369–386. doi: 10.1002/pits.20303

[ref6] Ashwin T. S. Guddeti R. M. R. M. (2020). Automatic detection of students’ affective states in classroom environment using hybrid convolutional neural networks. Educ. Inf. Technol. 25, 1387–1415. doi: 10.1007/s10639-019-10004-6

[ref7] Bierman K. L. Domitrovich C. E. Nix R. L. Gest S. D. Welsh J. A. Greenberg M. T.. (2008). Promoting academic and social-emotional school readiness: the head start REDI program. Child Dev. 79, 1802–1817. doi: 10.1111/j.1467-8624.2008.01227.x, PMID: 19037951 PMC3549580

[ref8] Blasco-Giner C. Battistelli A. Meneghel I. Salanova M. (2023). Psychological capital, autonomous motivation and innovative behavior: a study aimed at employees in social networks. Psychol. Rep. 13:332941231183614. doi: 10.1177/00332941231183614, PMID: 37311221

[ref9] Brown T. A. (2015). Confirmatory factor analysis for applied research. 2nd Edn. New-York, NY: Guilford Press.

[ref10] Byrne B. M. (1994). Structural equation modeling with EQS and EQS/windows. Thousand Oaks, CA: Sage Publications.

[ref11] Carmona-Halty M. Salanova M. Llorens S. Schaufeli W. B. (2021). Linking positive emotions and academic performance: the mediated role of academic psychological capital and academic engagement. Curr. Psychol. 40, 2938–2947. doi: 10.1007/s12144-019-00227-8

[ref12] Cerasoli C. P. Nicklin J. M. Ford M. T. (2014). Intrinsic motivation and extrinsic incentives jointy predict performance: a 40-year meta-analysis. Psychol. Bull. 140, 980–1008. doi: 10.1037/a0035661 24491020

[ref13] Cirak K. S. Yildirim I. Cucuk E. (2018). The effects of blended learning on student achievement: a Meta-analysis study. Hacettepe Univ. egitim Fakultesi Dergisi-Hacettepe Univ. J. Educ. 33, 1–27. doi: 10.16986/HUJE.2017034685

[ref14] Connell J. P. Spencer M. B. Aber J. L. (1994). Educational risk and resilience in African-American youth: context, self, action, and outcomes in school. Child Dev. 65, 493–506. doi: 10.2307/1131398 8013236

[ref15] Daniels L. M. Stupnisky R. H. Pekrun R. Hanyes T. L. Perry R. P. Newall N. E. (2009). A longitudinal analysis of achievement goals: from affective antecedents to emotional effects and achievement outcomes. J. Educ. Psychol. 101, 948–963. doi: 10.1037/a0016096

[ref16] Deci E. L. Olafsen A. H. Ryan R. M. (2017). Self-determination theory in work organizations: the state of a science. Annu. Rev. Organ. Psych. Organ. Behav. 4, 19–43. doi: 10.1146/annurev-orgpsych-032516-113108

[ref17] Dixson M. D. (2010). Creating effective student engagement in online courses: what do students find engaging? J. Scholar. Teach. Learn. 10, 1–13.

[ref18] Engels M. C. Spilt J. Denies K. Verschueren K. (2021). The role of affective teacher-student relationships in adolescents’ school engagement and achievement trajectories. Learn. Instr. 75:101485. doi: 10.1016/j.learninstruc.2021.101485

[ref19] Farrell A. M. (2009). Insufficient discriminant validity: a comment on Bove, Pervan, Beatty and Shiu. J. Bus. Res. 63, 324–327. doi: 10.1016/j.jbusres.2009.05.003

[ref20] Fischer C. Malycha C. P. Schafmann E. (2019). The influence of intrinsic motivation and synergistic extrinsic motivators on creativity and innovation. Front. Psychol. 10, 1–15. doi: 10.3389/fpsyg.201900137 30778313 PMC6369195

[ref21] Fornell C. Larcker D. F. (1981). Evaluating structural equation models with unobservable variables and measurementerror. J. Mark. Res. 18, 39–50. doi: 10.1177/002224378101800104

[ref22] Fredricks J. A. Blumenfeld P. C. Paris A. H. (2004). School engagement: potential of the concept state of the evidence. Rev. Educ. Res. 74, 59–109. doi: 10.3102/00346543074001059

[ref23] Fredricks J. A. McColskey W. (2012). “The measurement of student engagement: a comparative analysis of various methods and student self-report instruments” in Handbook of research on student engagement. eds. Christenson S. L. Reschly A. L. Wylie C. (Boston, MA: Springer US), 763–782.

[ref24] Fu L. Qiu Y. (2023). Contributions of psychological capital to the learning engagement of Chinese undergraduates in blended learning during the prolonged COVID-19 pandemic: the mediating role of learning burnout and the moderating role of academic buoyancy. Eur. J. Psychol. Educ. 7. doi: 10.1007/s10212-023-00759-5

[ref25] Ganotice F. A. Jr. Chan C. S. Chan E. W. Chan S. K. W. Chan L. Chan S. C. S.. (2022). Autonomous motivation predicts students' engagement and disaffection in interprofessional education: scale adaptation and application. Nurse Educ. Today 119:105549. doi: 10.1016/j.nedt.2022.105549, PMID: 36182789

[ref26] Gao B. W. Jiang J. Tang Y. (2020). The effect of blended learning platform and engagement on students' satisfaction-the case from the tourism management teaching. J. Hosp. Leis. Sport Tour. Educ. 27:100272. doi: 10.1016/j.jhlste.2020.100272

[ref27] Geertshuis S. A. (2019). Slaves to our emotions: examining the predictive relationship between emotional well-being and academic outcomes. Act. Learn. High. Educ. 20, 153–166. doi: 10.1177/1469787418808932

[ref28] Guthrie J. T. Wigfield A. You W. (2012). “Instructional contexts for engagement and achievement in reading” in Handbook of research on student engagement. Boston. eds. Christenson S. L. Reschly A. L. Wylie C. (Boston, MA: Springer US), 601–634.

[ref29] Hair J. F. Black W. C. Babin B. J. Anderson R. E. (2010). Multivariate data analysis. 7th Edn. New Jersey, NJ: Pearson.

[ref30] Halverson L. R. Graham C. R. (2019). Learner engagement in blended learning environments: a conceptual framework. Online Learn. 23, 145–178. doi: 10.24059/olj.v23i2.1481

[ref31] Hasnain N. Hasan Z. Chorath S. (2017). Organizational citizenship behavior and work motivation as correlates of psychological capital among public and private school teachers. Int. J. Soc. Sci. Educ. Stud. 3:133. doi: 10.23918/ijsses.v3i3p133

[ref32] Hasnine M. N. Nguyen H. T. Tran T. T. T. Bui H. T. Akçapınar G. Ueda H. (2023). A real-time learning analytics dashboard for automatic detection of online learners’ affective states. Sensors 23:4243. doi: 10.3390/s23094243, PMID: 37177447 PMC10181135

[ref33] Hodges C. B. (2004). Designing to motivate: motivational techniques to incorporate in e-learning experiences. J. Interact. Online Learn. 2, 1–7, Available at: http://www.ncolr.org/jiol/issues/pdf/2.3.1.pdf

[ref34] Hu L. T. Bentler P. M. (1999). Cutoff criteria for fit indexes in covariance structure analysis: conventional criteria versus new alternatives. Struct. Equ. Model. Multidiscip. J. 6, 1–55. doi: 10.1080/10705519909540118

[ref35] Jafri M. H. (2013). A study of the relationship of psychological capital and students' performance. Bus. Perspect. Res. 1, 9–16. doi: 10.1177/2278533720130202

[ref36] Kahu E. R. (2013). Framing student engagement in higher education. Stud. High. Educ. 38, 758–773. doi: 10.1080/03075079.2011.598505

[ref37] Khajavy G. H. Makiabadi H. Navokhi S. A. (2019). The role of psychological capital in language learners’ willingness to communicate, motivation, and achievement. Eurasian J. Appl. Linguist. 5, 495–513. doi: 10.32601/ejal.651346

[ref38] King R. B. Caleon I. S. (2021). School psychological capital: instrument development, validation, and prediction. Child Indic. Res. 14, 341–367. doi: 10.1007/s12187-020-09757-1

[ref39] Kobicheva A. Tokareva E. Baranova T. (2022). Students’ affective learning outcomes and academic performance in the blended environment at university: comparative study. Sustain. For. 14:11341. doi: 10.3390/su141811341

[ref40] LaRocca M. A. Marshall D. R. Groves K. S. (2023). Exploring the motivation to lead in a demanding environment: the role of achievement values, grit, and psychological capital. Psychol. Rep. 1–24. doi: 10.1177/00332941231199456 37643627

[ref41] Lazowski R. A. Hulleman C. S. (2016). Motivation interventions in education: a meta-analytic review. Rev. Educ. Res. 86, 602–640. doi: 10.3102/0034654315617832

[ref42] Lee Y. Capraro R. M. Capraro M. M. Bicer A. (2022). Cultural affordance, motivation, and affective mathematics engagement in Korea and the US. Math. Educ. Res. 25, 21–43. doi: 10.7468/jksmed.2022.25.1.21

[ref43] Lester D. (2013). A review of the student engagement literature. Focus Colleg. Univ. Schools. 7, 1–8.

[ref44] Liew J. McTigue E. Barrois L. Hughes J. (2008). Adaptive and effortful control and academic self-efficacy beliefs on achievement: a longitudinal study of 1 through 3 graders. Early Child Res. Q. 23, 515–526. doi: 10.1016/j.ecresq.2008.07.003, PMID: 19169387 PMC2630289

[ref45] Liu S. Liu S. Liu Z. Peng X. Yang Z. (2022). Automated detection of emotional and cognitive engagement in MOOC discussions to predict learning achievement. Comput. Educ. 181:104461. doi: 10.1016/j.compedu.2022.104461

[ref46] Luthans F. Avolio B. J. Avey J. B. Norman S. M. (2007a). Positive psychological capital: measurement and relationship with performance and satisfaction. Pers. Psychol. 60, 541–572. doi: 10.1111/j.1744-6570.2007.00083.x

[ref47] Luthans F. Youssef-Morgan C. M. (2017). Psychological capital: an evidence-based positive approach. Annu. Rev. Organ. Psych. Organ. Behav. 4, 339–366. doi: 10.1146/annurev-orgpsych-032516-113324

[ref48] Luthans F. Youssef-Morgan C. M. Avolio B. J. (2007b). Psychological capital: Developing the human competitive edge. Oxford: Oxford University Press.

[ref49] Manwaring K. C. Larsen R. Graham C. R. Henrie C. R. Halverson L. R. (2017). Investigating student engagement in blended learning settings using experience sampling and structural equation modeling. Internet High. Educ. 35, 21–33. doi: 10.1016/j.iheduc.2017.06.002

[ref50] Maroco J. Maroco A. L. Campos J. A. D. B. Fredricks J. A. (2016). University student’s engagement: Development of the university student engagement inventory (USEI). Brazil: Psicologia: Reflexão e Crítica.

[ref51] Mehta N. K. Prasad S. S. Saurav S. Saini R. Singh S. (2022). Three-dimensional DenseNet self-attention neural network for automatic detection of student’s engagement. Appl. Intell. 52, 13803–13823. doi: 10.1007/s10489-022-03200-4, PMID: 35340984 PMC8932470

[ref52] Merriam S. B. Bierema L. L. (2013). Adult learning: Linking theory and practice. Hoboken, NJ: John Wiley & Sons.

[ref53] Nunnally J. C. Bernstein I. H. (1994). Psychometric theory. 3rd Edn. New York, NY: McGraw-Hill.

[ref54] Peng R. Z. Fu R. R. (2021). The effect of Chinese EFL students' learning motivation on learning outcomes within a blended learning environment. Australasian Soc. Comput. Learn. Tertiary Educ.-Ascilite 37, 61–74. doi: 10.14742/ajet.6235

[ref55] Pintrich P. R. Schunk D. H. (1996). Motivation in education: Theory, research, and applications. Englewood Cliff, N.J: Merrill.

[ref56] Pöysä S. Vasalampi K. Muotka J. Lerkkanen M. K. Poikkeus A. M. Nurmi J. E. (2018). Variation in situation-specific engagement among lower secondary school students. Learn. Instr. 53, 64–73. doi: 10.1016/j.learninstruc.2017.07.007

[ref57] Ramirez-Arellano A. Bory-Reyes J. Hernández-Simón L. M. (2019). Emotions, motivation, cognitive–metacognitive strategies, and behavior as predictors of learning performance in blended learning. J. Educ. Comput. Res. 57, 491–512. doi: 10.1177/0735633117753935

[ref58] Ravat S. Barnard-Ashton P. Keller M. M. (2021). Blended teaching versus traditional teaching for undergraduate physiotherapy students at the university of the Witwatersrand. South African. J. Physiother. 77:1544. doi: 10.4102/sajp.v77i1.1544, PMID: 34192211 PMC8182468

[ref59] Raver C. C. (2002). Emotions matter: making the case for the role of young children’s emotional development for early school readiness. Soc. Policy Rep. 16, 1–20. doi: 10.1002/j.2379-3988.2002.tb00041.x

[ref60] Raykov T. Marcoulides G. A. (2011). Introduction to psychometric theory. New York, NY: Routledge.

[ref61] Reeve J. (2012). “A self-determination theory perspective on student engagement” in Handbook of research on student engagement. eds. Christenson S. L. Reschly A. L. Wylie C. (Boston, MA: Springer US).

[ref62] Schunk D. H. Pintrich P. R. Meece J. L. (2008). Motivation in education: Theory, research, and applications. Upper Saddle River, NJ: Merrill Prentice Hall.

[ref63] Siu O. L. Bakker A. B. Jiang X. (2014). Psychological capital among university students: relationships with study engagement and intrinsic motivation. J. Happiness Stud. 15, 979–994. doi: 10.1007/s10902-013-9459-2

[ref64] Skinner E. Furrer C. Marchand G. Kindermann T. (2008). Engagement and disaffection in the classroom: part of a larger motivational dynamic? J. Educ. Psychol. 100, 765–781. doi: 10.1037/a0012840

[ref65] Skinner E. A. Kindermann T. A. Furrer C. J. (2009). A motivational perspective on engagement and disaffection: conceptualization and assessment of children's behavioral and emotional participation in academic activities in the classroom. Educ. Psychol. Meas. 69, 493–525. doi: 10.1177/0013164408323233

[ref66] Slåtten T. Lien G. Batt-Rawden V. H. Evenstad S. B. N. Onshus T. (2023). The relationship between students’ psychological capital, social-contextual factors and study-related outcomes–an empirical study from higher education in Norway. Int. J. Qual. Serv. Sci. 15, 17–33. doi: 10.1108/IJQSS-11-2021-0160

[ref67] Slavin R. E. (2005). Educational psychology: Theory and practice (8th Edition). Boston: Allyn & Bacon.

[ref68] Tripon C. Gonța I. Bulgac A. (2023). Nurturing minds and sustainability: an exploration of educational interactions and their impact on student well-being and assessment in a sustainable university. Sustain. For. 15:9349. doi: 10.3390/su15129349

[ref69] Trowler P. Trowler V. (2010). Student engagement evidence summary. Lancaster: Lancaster University. Available at: http://eprints.Lancs.Ac.Uk/61680/1/deliverable._2_Evidence_Summary._Nov_2010.Pdf.

[ref70] Wang Z. Bergin C. Bergin D. A. (2014). Measuring engagement in fourth to twelfth grade classrooms: the classroom engagement inventory. Sch. Psychol. Q. 29, 517–535. doi: 10.1037/spq0000050, PMID: 24708283

[ref71] Wang M. T. Willett J. B. Eccles J. S. (2011). The assessment of school engagement: examining dimensionality and measurement invariance by gender and race/ethnicity. J. Sch. Psychol. 49, 465–480. doi: 10.1016/j.jsp.2011.04.001, PMID: 21724001

[ref72] Wimpenny K. Savin-Baden M. (2013). Alienation, agency, and authenticity: a synthesis of the literature on student engagement. Teach. High. Educ. 18, 311–326. doi: 10.1080/13562517.2012.725223

[ref73] Witkowski P. Cornell T. (2015). An investigation into student engagement in higher education classrooms. J. Scholar. Teach. 10, 56–67. doi: 10.46504/10201505wi

[ref74] Yin B. Yuan C.-H. (2021). Precision teaching and learning performance in a blended learning environment. Front. Psychol. 12:631125. doi: 10.3389/fpsyg.2021.631125, PMID: 33613404 PMC7892782

[ref75] Yu Z. Xu W. Sukjairungwattana P. (2022). Meta-analyses of differences in blended and traditional learning outcomes and students' attitudes. Front. Psychol. 13:926947. doi: 10.3389/fpsyg.2022.926947, PMID: 36186290 PMC9524290

